# Contraceptive practices amongst HIV-positive women on antiretroviral therapy attending an ART clinic in South Africa

**DOI:** 10.4102/phcfm.v5i1.461

**Published:** 2013-05-08

**Authors:** Ezekiel E. Oni, Andrew Ross, Stephan van der Linde

**Affiliations:** 1 Department of Family Medicine, University of KwaZulu-Natal, South Africa; 2 Department of Public Health, University of KwaZulu-Natal, South Africa

## Abstract

**Background:**

Effective contraceptive practices amongst HIV-positive women of reproductive age have been shown to reduce mother-to-child transmission of HIV by preventing unplanned pregnancies. However, most antiretroviral therapy (ART) programmes focus on treatment, neglecting comprehensive contraceptive services. This results in a high frequency of pregnancies amongst HIV-positive women attending the ART clinic of a regional hospital north of Durban.

**Objectives:**

This research aimed to explore contraceptive use amongst HIV-positive women attending an ART clinic by determining, (1) prevalence of contraceptive use, (2) pregnancy rate, (3) contraceptive preferences and (4) factors associated with contraceptive use.

**Methods:**

In this observational, analytical, cross-sectional study of 420 women, aged 15 to 49 years, participants were selected by systematic random sampling. They completed standardised questionnaires.

**Results:**

Of all participants, 95% of the participants used contraception. Factors associated with contraceptive practice were knowledge of HIV status 292 (72.8%), health worker advice 84 (20.9%), and spousal insistence 33 (8.2%). Of the 130 women (31%) who had fallen pregnant whilst on ART, 73 (56.2%) said that the pregnancy had been unplanned, whilst 57 (43.8%) had wanted to fall pregnant because of: partner's insistence (45.6%), desire for a child (36.8%), desire to conceal HIV status (15.8%), not wanting to die childless (5.3%), and death of a previous child (1.8%).

**Conclusion:**

Contraceptive use amongst these women was high but the number of pregnancies is a cause for concern. Information regarding contraceptive use should therefore be provided at all ART clinics.

## Abstrait

**Les pratiques contraceptives parmi les femmes séropositives sous traitement antirétroviral fréquentant une clinique ART en Afrique du Sud**

**Présentation:**

Il a été prouvé que les bonnes pratiques contraceptives chez les femmes séropositives en âge de procréer réduisaient la transmission du VIH de la mère à l'enfant par la prévention des grossesses non planifiées. Mais la majorité des programmes de thérapie antirétrovirale (ARV) se concentrent sur le traitement, au détriment de services complets axés sur la contraception. La conséquence en est une fréquence élevée de grossesses chez les femmes séropositives fréquentant le centre de traitement aux ARV d'un hôpital régional du Nord de Durban.

**Objectifs:**

L'objectif de cette étude était de se pencher sur l'utilisation de contraceptifs chez les femmes séropositives fréquentant un centre de traitement aux ARV en déterminant, (1) la prévalence du recours aux contraceptifs, (2) le taux de grossesses, (3) les préférences en matière de contraception and (4) les facteurs associés à l'utilisation de contraceptifs.

**Méthodes:**

Dans cette étude d'observation, analytique et transversale réalisée auprès de 420 femmes, âgées de 15 à 49 ans, les participantes ont été sélectionnées par échantillonnage aléatoire systématique. Elles ont répondu à des questionnaires standardisés.

**Résultats:**

The résultats 95% des participantes avaient recours à la contraception. Les facteurs associés à la pratique contraceptive étaient la connaissance du statut sérologique 292 (72,8%), les conseils des travailleurs de la santé 84 (20,9%) et l'insistance du conjoint 33 (8,2%). Sur les 130 femmes (31%) qui étaient tombées enceintes alors qu'elles étaient sous ARV, 73 (56,2) ont affirmé que la grossesse n'avait pas été planifiée, et 57 (43,8%) avaient souhaité tomber enceinte pour les raisons suivantes: insistance du partenaire (45,6%), désir d'enfant (36,8%), volonté de cacher son statut sérologique (15,8%), ne pas mourir sans avoir eu d'enfant (5,3%) et décès d'un enfant précédent (1,8%).

**Conclusion:**

L'utilisation de contraceptifs chez ces femmes était élevée, mais le nombre de grossesses est inquiétant. Des informations sur l'utilisation des contraceptifs devraient par conséquent être fournies dans tous les centres de traitement aux ARV.

## Introduction

Women account for half of the estimated 31.3 million adults living with HIV and AIDS worldwide, the majority of whom are in their reproductive years.^1, 2, 3^ Globally more than 2 million HIV-positive women fall pregnant each year,^4^ with up to 600 000 dying of pregnancy-related complications annually, mostly in resource-constrained settings.^4^ Many HIV-infected women are sexually active^5^ and increase or resume sexual activity as their health improves whilst on antiretroviral therapy (ART).^6^ Studies from across Africa show that many pregnancies are unplanned, and that maternal deaths can be avoided if correct and consistent contraceptive use is promoted amongst women of childbearing age living with HIV and AIDS. South Africa's fourth confidential enquiries into maternal deaths showed that AIDS accounted for the highest number of maternal deaths arising from non-pregnancy related infections.^7^ However, available data on contraceptive practices amongst women of childbearing age living with HIV show a (generally) low level of usage, particularly in Africa.^8^

Most children with HIV were infected by mother-to-child transmission (MTCT) of the virus.^9^ Across Africa, up to 1 900 children are infected daily with HIV, and three million children younger than 15 are living with the disease.^10^ Effective contraception has been shown to reduce MTCT of HIV by preventing unwanted pregnancies.^11^ It is estimated that approximately 120 000 HIV-positive births per year would be averted if the family planning needs of all HIV-positive women in sub-Saharan Africa could be met.^11^ However, most antiretroviral therapy (ART) programmes focus on providing ART to HIV-positive women without integration with contraceptive services.^12^

Most methods of contraception can be used by women who are HIV-positive,^13^ and correct and consistent use of condoms can prevent pregnancies and STI.^13^ The effectiveness of low-dose hormonal contraceptives may be reduced by ART, which accelerates the breakdown of the hormones, necessitating the use of condoms to compensate for this.^13^ Intrauterine devices can be inserted if HIV-positive women are clinically stable and on ART.^13^ Diaphragms and cervical caps are not recommended by WHO because of their use of spermicides which contain nonoxynol-9,^14^ which is reported to cause epithelial damage, thereby increasing the risk of STI and HIV when used frequently.^15^ Sterilisation is an excellent option for HIV-positive women who have completed their families, but barrier methods would still need to be used to protect against STI.^13^

A partner's HIV status has been shown in several studies to influence contraceptive practices amongst women of childbearing age living with HIV or AIDS. A study performed in France revealed that 91% of women in a serodiscordant relationship used contraception compared to 69% of women in a seroconcordant relationship.^16^ Consistent condom use has been found to be higher in women whose sexual partners were HIV negative, whilst the use of oral contraceptive pills and intrauterine devices was higher in seroconcordant couples.^16^ A study performed in Cape Town between May 2004 and January 2005 which examined reproductive intentions amongst HIV-positive women and men, revealed that factors such as personal, intimate partner relations as well as social factors were important in determining respondents’ reproductive intentions, and by extension, contraceptive practices.^17^ In the Cape Town study personal desire for biological parenthood were found to be stronger than their HIV-seropositive status in influencing women's decision on contraceptive practices.^17^

In a study in Uganda the authors concluded that a strong desire for children may be responsible for the low acceptance of contraceptive methods amongst the HIV-positive respondents.^18^ HIV-positive women considered having children as a way of showing their ‘normal’ status and having their own child was seen as bringing hope, happiness, and a reason to live for HIV-positive women.^17^ Another important factor affecting contraceptive use was strong partner expectations for children, with some women feeling compelled to abandon contraception and get pregnant for fear of infidelity and abandonment.^17^ A study performed in Burkina Faso between 1995 and 1999 to describe the sexual and reproductive life of women informed of their HIV-seropositive status, revealed that an adverse event i.e. the death of a previous child, was the only predictor of subsequent pregnancies amongst study participants.^19^ There is little published data on the contraceptive knowledge and practice amongst HIV-positive women attending ART clinics in KwaZulu–Natal.

## Aim of the study

The aim of this research was therefore to explore contraceptive practices amongst women of childbearing age living with HIV or AIDS attending the ART clinic at a large regional hospital north of Durban.

## Ethical considerations

Ethical approval was obtained from the Biomedical Research Ethics Committee of the University of KwaZulu-Natal, hospital management and the Department of Health. Written consent was obtained from all study participants after a detailed explanation of the purpose of the study.

### Potential benefits and hazards

Study participants were told that part of the benefits to be derived from the research would be a better understanding of contraception as it relates to women living with HIV or AIDS, and that the information gathered from the study would help healthcare personnel to appreciate the difficulties being faced by patients as regards contraceptive practice so that better contraceptive care could be provided. They were told that there were no risks involved in participating in the research and that any information given by them would not be used against them.

### Recruitment procedures

Participants were advised that participation was entirely voluntary. They were told that they reserved the right to decide whether or not to participate, and that they might withdraw from the study at any time without giving a reason.

### Informed consent

Participants received explanations on what the study entailed in a language they could understand. They were given the opportunity to ask questions and thereafter a signed consent was obtained from them.

### Data protection

Participants were advised against giving their names, and their identities were not documented. This was performed to ensure confidentiality.

## Methods

### Materials

The study included HIV-positive women 15 to 49 years of age attending the ART clinic. Patients completed a standardised questionnaire adapted from a previously validated questionnaire^20^ which had been piloted with ten female patients attending the primary health care clinic. The questionnaire consisted of two parts, Part 1 dealing with socio-demographic characteristics and Part 2 exploring contraceptive practices.

### Context of the study

The study was performed at the ART clinic at Ngwelezane Hospital, a large regional hospital north of Durban. The clinic provides services to predominantly African patients who live in the surrounding urban and rural areas where unemployment is high and many people rely on social grants. Approximately 2500 women of childbearing age attend the ART clinic on a monthly basis. Between October 2011 and December 2011, 420 women (representing 20% of the study population) were selected using a systematic random sampling method.

### Design

This was an observational, analytical, cross-sectional study.

### Procedure

All HIV-positive women between 15 and 49 years of age attending the HIV clinic were considered part of the study population. A systematic random sampling method was used in selecting study participants who met the inclusion criteria. A number between one and three was randomly selected by the researcher and then, starting with this number, every third patient who met the inclusion criteria was chosen. Participants who had been chosen were informed of the details of the study and the content of the patient information booklet was explained in a language understandable to them, mostly isiZulu. They were encouraged to ask questions to address whatever concerns they had. Thereafter a signed consent was obtained from them, and the process of completing the questionnaires initiated. All this was performed on individual basis.

### Analysis

Data were entered into a Microsoft Office Excel spreadsheet and analysed using SPSS version 19.0 (SPSS Inc., Chicago, Illinois). Data were analysed descriptively. A *p*-value of <0.05 was considered to be significant.

## Results

The women included in the study were aged between 15 and 49 years, with a median age of 32 years. Of the participants 99% (416/420) were Africans, 70% (281/420) were unemployed and more than half had two or more children whilst more than 80% were single (Table 1). Seventy five percent had been on ART for three years or less.

**TABLE 1:** Demographic characteristics of the study population.

Variable	Category	*n*	%
Level of education	No schooling	16	3.8
	Primary	49	11.7
	Secondary	307	73.1
	Tertiary	48	11.4
Parity	No children	50	11.9
	1 child	131	31.2
	2 or more children	239	56.9
Marital status	Single	344	81.9
	Married	69	16.4
	Divorced	2	0.5
	Separated	2	0.5
	Widow	3	0.7
ART duration	< 1 year	150	35.7
	2 years	103	24.5
	3 years	69	16.4
	4 years	32	7.6
	5 years	32	7.6
	➣ 5 years	34	8.1

*n*, Given as number.

Forty-two percent of the women (177/420) did not know their partner's HIV status, 46.4% (195/420) said that their partners were HIV-positive and 11.4% (48/420) knew that their partners were HIV-negative (Table 2).

**TABLE 2:** Partner's HIV status and current condom use.

Partner HIV status	Current condom usage					
	
	No		Yes		Total	
			
	*n*	%	*n*	%	*n*	%
Negative	14	8	34	13.8	48	11.4
Positive	76	43.7	119	48.4	195	46.4
Unknown	84	48.3	93	37.8	177	42.0

**Total**	**174**	**41.4**	**246**	**58.6**	**420**	-

*n*, Given as number.

Ninety-six percent of the study participants (401/420) indicated that they were currently using contraceptives. Of these 61.3% were using condoms (alone or combined with other methods), 20.0% injectable contraceptive, 19.7% abstinence, 4.5% intrauterine contraceptive device (IUCD), 2.2% pills and 0.7% coitus interruptus (Table 3). A combination of condoms and injectable contraceptives was reported by 27 participants, a combination of condoms and pills was reported by four participants, whilst three study participants reported using a combination of condoms and IUCD. Condom use was highest amongst patients in serodiscordant relationships (71%), and lowest amongst patients whose partners’ HIV status was unknown (61%). Factors associated with contraceptive practice were: knowledge of HIV status 292 (72.8%), health worker advice 84 (20.9%), and spousal insistence 33 (8.2%).

**TABLE 3:** Current and previous contraceptive use.

Contraceptive method	Current contraceptive use		Previous contraceptive use		*p*-value
			
	*n*	%	*n*	%	
Condoms	246	61.3	27	6.4	*<* 0.001
Injection	80	20.0	267	64.0	0.004
Pills	9	2.2	31	7.4	0.137
IUCD	18	4.5	1	0.2	0.832
Abstinence	79	19.7	1	0.2	0.63
Coitus interruptus	3	0.7	10	2.4	*<* 0.001
No contraceptive	19	-	-	-	-

IUCD, intrauterine contraceptive device. *n*, Given as number.

Study participants showed significant changes in the contraceptive methods used before and after HIV diagnosis. Significantly, more women used condoms after HIV diagnosis or initiation of ART: 246 (61.3%) compared to 27 (6.4%) prior to HIV diagnosis (*p* < 0.001). Fewer used contraceptive injections after diagnosis: 80 (20.0%) compared to 267 (64.0%) prior to HIV diagnosis (*p* = 0.004) (Table 3). Nineteen participants were not using any contraceptives at all.

Contraceptive use was higher amongst the patients with some level of education, with 389/404 (96.3%) using a contraceptive method compared to those with no education, 16, amongst whom 12 (75%) did use some form of contraception; *p =* 0.004 (Table 4).

**TABLE 4:** Contraceptive practice: educated versus uneducated participants.

Education	Variable	Not using contraceptive		Using contraceptive		Total (*n*)
				
		*n*	%	*n*	%	
Educational status	None	4	25	12	75	16
	Some	15	3.7	389	96.3	404
	**Total**	**19**	**4.5**	**401**	**95.5**	**420**
Educational level	None	4	25	12	75	16
	Primary	2	-	47	-	49
	Secondary	9	-	298	-	307
	Tertiary	4	-	44	-	48
	**Total**	**19**	**4.5**	**401**	**95.5**	**420**

*n*, Given as number.

One hundred and thirty (31%) women reported having been pregnant since commencement of ART; 90% had been pregnant once, and two had been pregnant on more than two occasions. Of the 130 women who reported pregnancies, 73 (56.2%) said that the pregnancies had been unplanned, whilst 57 (43.8%) had desired the pregnancies. For those who had intended to fall pregnant, the main reasons for doing so were their partner's insistence, 26 (45.6%), and a strong desire for a child (36.8%). Other reasons given are listed below in Table 5.

**TABLE 5:** Pregnancy status and reasons of those falling pregnant.

Pregnancy since on ART	Variable	*n*	%
Number of pregnancies	1	117	90.00
	2	11	8.50
	3	2	1.50
	**Total**	**130**	-
Intentions regarding pregnancy	Not planned	73	56.20
	Desired pregnancy	57	43.80
Reasons for desired pregnancy	Partner's insistence	26	45.60
	Wanted a child	21	36.80
	Conceals HIV status	9	15.80
	Not wanting to die childless	3	5.30
	Death of previous child	1	1.80

*n*, Given as number.

## Discussion

The demographics in this study are consistent with those who are known to be at greatest risk of HIV infection - the young and sexually active. The majority of the participants had attained some level of education, and despite many having at least two children, more than 80% were single, suggesting that partnerships were not long-term, something which has implications for an increased risk of HIV transmission.

It is of concern that the HIV status of partners is not known by a large percentage of the study participants and suggests that intimate matters such as HIV status are not being discussed. It may be a result of problems associated with disclosure and its attendant risks. Male partners who are already aware of their HIV-seropositive status may deliberately withhold such information from their female partners to maintain their dominance in the relationship.

The prevalence of contraceptive practice was higher (95.5%) than that reported in previous studies amongst HIV-positive women on ART. The African DITRAME study in Abidjan, Cote d'Ivoire, for instance, reported that only 39% of HIV-positive women used contraceptives.^21^ The higher contraceptive practice amongst these study participants may be due to the active promotion of contraceptives during the pre-initiation counselling classes and subsequent follow-up visits. This is reflected in the large number (58.6%) who opted for condoms as their preferred contraceptive choice after being diagnosed with HIV, in comparison to the relatively small number of those using condoms (6.4%) prior to the initiation of ART. In the DITRAME study, the level of education was found to be significantly associated with contraceptive use,^21^ which is consistent with the findings of this study. Of the participants 96% have some education and this may be another reason for the higher contraceptive use found in this study. Condom use was also highest amongst participants in serodiscordant relationships, consistent with findings in the French study.^16^ Knowledge of HIV status, health worker advice, and spousal insistence were identified in this study as important factors associated with contraceptive practice. These findings are consistent with factors identified in the study in Cape Town.^17^ Despite the fairly high level of contraceptive practice amongst the study participants, the use of dual methods was extremely low.

The 31% pregnancy rate in this study, with 56.2% indicating that the pregnancy was unplanned, is consistent with a similar study performed in Zimbabwe in 1999 involving 59 HIV-positive women of whom 18 (31%) fell pregnant despite knowing their seropositive status.^22^ Other studies have shown much higher pregnancy rates. A study performed in Uganda in 2005 reported that 96% of the 84 study participants who experienced pregnancies said they had been unplanned.^23^ These high frequencies of unplanned pregnancies reveal an unmet need for contraception and also highlights some of the challenges women face when using condoms as the preferred method of contraception. These include women fearing disclosure of their HIV seropositive status, their inability to decide or insist on condom use during intercourse on account of their culturally weak position, and accidental breakage or slippage during intercourse.^24^ Some reports have shown condom accident rate to range from 1% – 12% and its failure rate at least 12%.^25, 26^ The high rate of unintended pregnancies in our study further highlights the importance of promoting dual protection (condom plus another form of contraceptive) as a way of ensuring a reliable contraceptive method to provide extra protection against pregnancy beyond that which condom use alone provides.

A study in Cape Town showed that a personal desire for biological parenthood was more likely to influence a women's decision on contraceptive practices than the fear of transmitting the HIV.^17^ For those who desired pregnancy, the following were identified as reasons: partner's insistence, strong desire for a child, to conceal HIV-seropositive status, not wanting to die childless, and death of a previous child. This is similar to the finding of wanting to appear uninfected in the Cape Town study,^17^ as well as to the Burkina Faso study, where death of a previous child was an important motivation.^19^

It is important for health care workers to advocate consistent use of dual protection if there are to be significant reductions in the burden of disease posed by HIV and AIDS. Women planning to have a baby must ensure a low viral load and a rising CD4 count to minimize the chances of MTCT of the disease.^27, 28^ Provision of effective contraception to prevent unintended pregnancies amongst HIV-positive women remains a key element in the UN Glion call to action on family planning and HIV and/or AIDS in women and children.^29^ Several models have shown the cost-effectiveness of the correct and consistent use of contraceptives; for the same expenditure, increasing contraceptive use amongst HIV-positive women will prevent more HIV-positive births than the traditional method of PMTCT with ART.^30, 31^ A study conducted in eight African countries revealed that the same number of paediatric HIV infections prevented by providing nevirapine to all HIV-positive women giving birth could be achieved at much lower cost by moderate reductions in the number of unintended pregnancies, ranging from approximately 6% in Kenya to 35% in Rwanda.^31^

### Limitation of the study

Generalisability of the results may be limited by the fact that study participants were predominantly African and may not be representative of a multi-racial society. Furthermore the study was performed at only one ART clinic at a regional hospital.

### Recommendations

Recommendations from this study include the integration of family planning services into ART programmes for HIV-positive women, and an emphasis on correct and consistent use of available contraceptive methods. A vigorous campaign should be made to advocate the adoption of dual protection as a way of not just reducing horizontal transmission of HIV and other STIs, but also reducing vertical transmission of HIV. Dual methods provide extra protection against pregnancy beyond the ability of condom use only to do so.

## Conclusion

This study has reported a good rate of condom use amongst HIV-positive women, a high rate of pregnancy and a low rate of dual contraceptive use, indicating an unmet need for effective contraception amongst HIV-positive women. Adequately addressing the contraceptive needs of these patients would serve a major public health purpose by reducing the burden of disease posed by HIV and AIDS.
